# Characterization of a Mass-Produced SiPM at Liquid Nitrogen Temperature for CsI Neutrino Coherent Detectors

**DOI:** 10.3390/s22031099

**Published:** 2022-01-31

**Authors:** Fang Liu, Xiaoxue Fan, Xilei Sun, Bin Liu, Junjie Li, Yong Deng, Huan Jiang, Tianze Jiang, Peiguang Yan

**Affiliations:** 1 Beijing Key Laboratory of Passive Safety Technology for Nuclear Energy, School of Nuclear Science and Engineering, North China Electric Power University, Beijing 102206, China; liuf@ncepu.edu.cn (F.L.); Fanxx@ncepu.edu.cn (X.F.); liu_bin@ncepu.edu.cn (B.L.); Jiangtz@ncepu.edu.cn (T.J.); 2 State Key Laboratory of Particle Detection and Electronics, Institute of High Energy Physics, Chinese Academy of Sciences, Beijing 100049, China; lijunjie@ihep.ac.cn (J.L.); dengyong@ihep.ac.cn (Y.D.); enginefly@163.com (H.J.); 3 Shenzhen Key Laboratory of Laser Engineering, College of Physics and Optoelectronic Engineering, Shenzhen University, Shenzhen 518060, China; yanpg@szu.edu.cn

**Keywords:** SiPM, breakdown voltage, dark count rate, liquid nitrogen temperature

## Abstract

Silicon Photomultiplier (SiPM) is a sensor that can detect low-light signals lower than the single-photon level. In order to study the properties of neutrinos at a low detection threshold and low radioactivity experimental background, a low-temperature CsI neutrino coherent scattering detector is designed to be read by the SiPM sensor. Less thermal noise of SiPM and more light yield of CsI crystals can be obtained at the working temperature of liquid nitrogen. The breakdown voltage (V_bd_) and dark count rate (DCR) of SiPM at liquid nitrogen temperature are two key parameters for coherent scattering detection. In this paper, a low-temperature test is conducted on the mass-produced ON Semiconductor J-Series SiPM. We design a cryogenic system for cooling SiPM at liquid nitrogen temperature and the changes of operating voltage and dark noise from room to liquid nitrogen temperature are measured in detail. The results show that SiPM works at the liquid nitrogen temperature, and the dark count rate drops by six orders of magnitude from room temperature (120 kHz/mm^2^) to liquid nitrogen temperature (0.1 Hz/mm^2^).

## 1. Introduction

Silicon photo-multipliers (SiPM) have been developed rapidly in recent years as an effective alternative for conventional Photo-multiplier Tubes (PMT). The SiPM sensor has many excellent characteristics [1], such as: compact size, easy to develop into detector arrays, works under the low bias voltage (V_bias_) and a strong ability to resist external magnetic and the electric field [2]. The key parameters, including the working voltage, dark count, quantum efficiency and gain for PMT and SiPM, are shown in Table 1 [3]. In addition, SiPM has high photon detection efficiency (PDE) and performs with an excellent single photon resolving ability. Due to these advantages, SiPM arrays are used for long-range high-speed light detection and the ranging (LiDAR) technique to achieve automotive, machine vision and spacecraft navigation [4,5]. The SiPM was used as read-out unit in the Circular Electron Positron Collider (CEPC) experiment for developing accurate measurements of the Higgs Boson [6], and the SiPM arrays were used to detect dark matter at liquid argon temperature [7]. The photon emission of SiPM from the avalanche pulses that were generated has been investigated in dark conditions [8].

The coherent elastic neutrino-nucleus scattering (CEνNS) method was first theorized by Freedman in 1974 [9,10] and was the dominated interaction for neutrinos in the energy range below 100 MeV. The COHERENT collaboration firstly detected the phenomenon of CEνNS by using CsI(Na) crystal detector to detect the neutrinos from the spallation neutron source at the Oak Ridge National Laboratory (ORNL) in 2017 [11,12]. The neutrinos produced from different neutrino sources presented different energy spectra, and as such the energy of the corresponding recoil nucleus from coherent scattering and the requirements for detection threshold and background are different [13]. The energy of neutrinos from the reactor source is in a low energy range, usually below 10 MeV, and the energy of the corresponding recoil nucleus is about a few keV. In addition, the count rate decreases exponentially as the detection threshold increases. Therefore, the threshold of the detector needs to be lower than 10 keV or less.

In order to achieve the low threshold detector, we propose a low temperature detector scheme using CsI crystals and SiPMs readout. CsI crystals have the highest light yield among mass produced crystals at low temperatures and can reach around 100 photons/keV [14]. However, the output signals of SiPM can effectively suppress its dark noise by controlling SiPM at low temperature, which can help to lower the detection threshold and obtain a high signal-to-noise ratio. The combination of CsI and SiPM can significantly reduce the detector threshold. Low temperatures can effectively suppress the shortcomings of the high dark count rate (DCR) of SiPMs. In older to reach the requirement of a low threshold detector, DCR should be lower than 0.1 Hz/mm^2^ at the liquid nitrogen temperature [15]. Selecting a SiPM that can work normally at the liquid nitrogen temperature is a key step in the detector scheme. It is best to be a mass-produced product for easy procurement. ON Semiconductor’s J-Series SiPM is a product widely used in the industry, and we have successfully applied it to the GECAM project [15].

In this study, we design a cryogenic system that can cool the SiPM measurement system to liquid nitrogen temperature and keep the SiPM working environment within a certain temperature range. In this research, we investigate this mass-produced SiPMs at liquid nitrogen temperature to determine whether it can be used in a coherent scattering experiment to detect neutrinos at low threshold.

## 2. Experiment Setup

**Figure 1:**
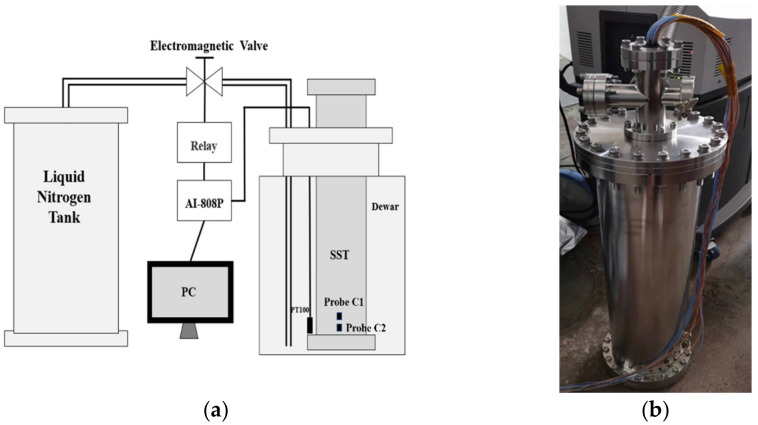
(**a**) Scheme of the cryogenic system; (**b**) photo of the stainless-steel chamber.

**Figure 2:**
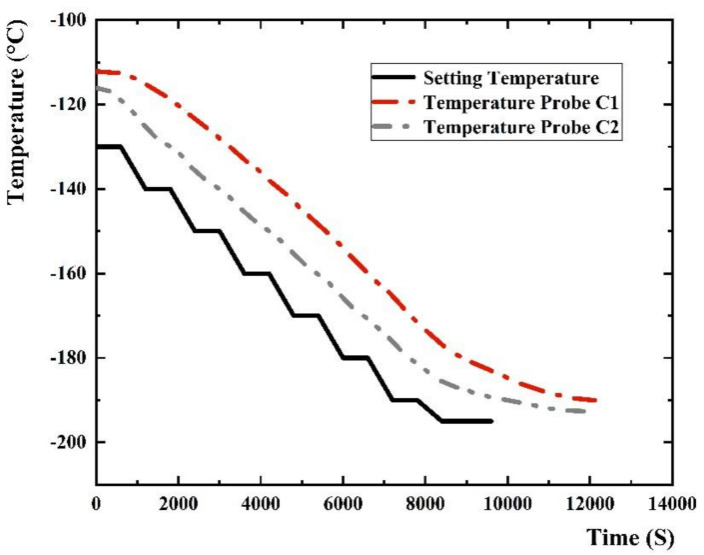
The temperature control and cooling curve of the system.

A set of self-made automatic temperature feedback control cryogenic systems is designed for cooling SiPM in an ease speed, because it will become brittle when the temperature of SiPM changes too fast, as shown in Figure 1a. It consists of a stainless-steel chamber, liquid nitrogen Dewar, temperature controller (AI-808P), temperature sensor, liquid nitrogen tank, solenoid valve and liquid nitrogen pipeline. We use three temperature sensors in this cryogenic system. One sensor (PT100) is placed at the outside bottom of the chamber to serve as the feedback of the temperature controller, and the other two (Probe C1 and C2, with distance of 5 cm) are put inside the chamber nearby SiPM to monitor the temperature in real time. The temperature controller, which adopts the proportional-integral-derivative (PID) control, controls the amount of liquid nitrogen entering the Dewar through the solenoid valve according to the set cooling rate. The photo of the stainless-steel chamber is shown in Figure 1b, with cable penetration and nitrogen replacement ports on the top. Before cooling down, the inside is replaced with nitrogen and sealed to avoid frost. The stable temperature keeping ability of the cryogenic system is important for the measurement accuracy. We measured the temperature retention ability of this system at the setting temperature 0 °C, and recorded the real-time change of temperature within 8000 s. The result indicates that the temperature is stable at 0 °C with a variation range from −0.3 °C to 0.3 °C. The temperature control and cooling curve of the system is shown in Figure 2 and the black solid curve is the setting temperature, with the dash line and dash-dot line showing the value of two temperature probes at different moments. The temperature changes at Probe C1 and C2 are gentle, about 0.5 degrees per minute, and finally reaching a stable plateau with temperature of C1 is −193 degrees, and C2 is −196 degrees. The temperature of C2 is close to the surface temperature of the SiPM circuit board, which represents the temperature of SiPM. The SiPM is J-60035, size 6 × 6 mm^2^ [16].

A Pico-ammeter (Keithley 2450) is used to measure the current versus voltage (I-V) curve of SiPM. In particular, at the liquid nitrogen temperature, an LED (500 nm) light source inside the top of the chamber is turned on; otherwise the breakdown voltage (V_bd_) of SiPM cannot be measured.

**Figure 3:**
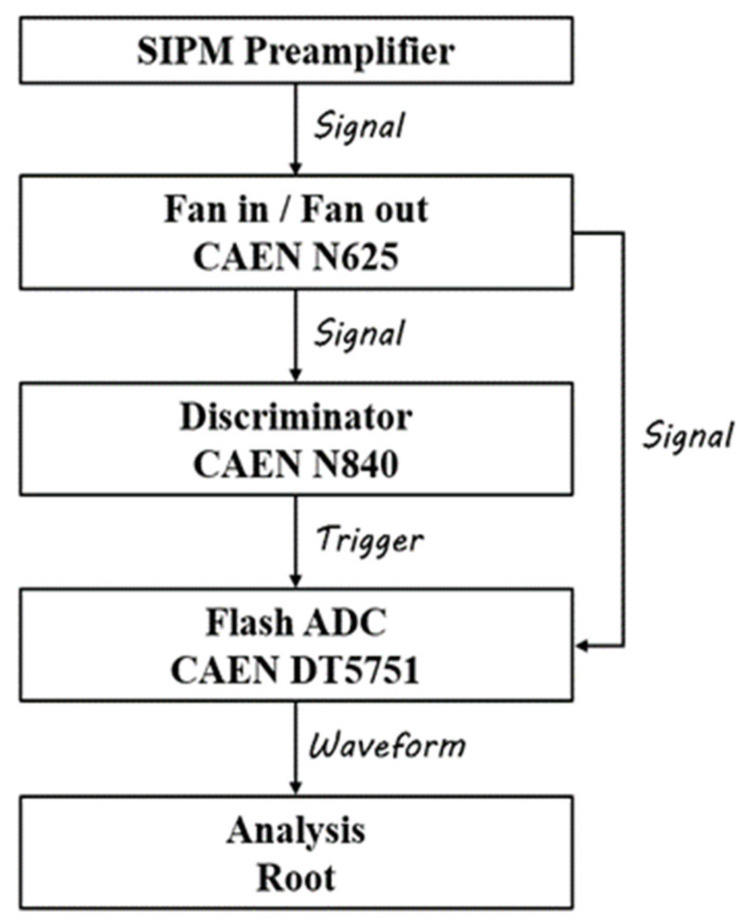
The dark noise measurement scheme.

The dark noise measurement scheme is shown in Figure 3. The SiPM signal enters the FIFO (fin in and fin out) (N625) [17] after preamplification, and one fan-out signal enters the low-threshold discriminator (N841) [18] for triggering of the data acquisition system (DT5751) [19], and another fan-out signal is directly connected to the data acquisition system. DT5751 has a counting mode and a waveform acquisition mode, which are used for DCR measurement and single photon spectrum measurement, respectively. The threshold for dark noise measurement is set to 0.5 single photoelectron. The over voltage of SiPM is kept unchanged when comparing noise measurements at different temperatures. The over voltage can be calculated as following:

(1) $$V_{\mathrm{over}}=V_{\mathrm{bias}}-V_{\mathrm{bd}}$$

where V_over_ is the over voltage, V_bias_ is the working voltage and V_bd_ is the breakdown voltage.

## 3. Measurements and Analysis

### 3.1. I-V Curve

**Figure 4:**
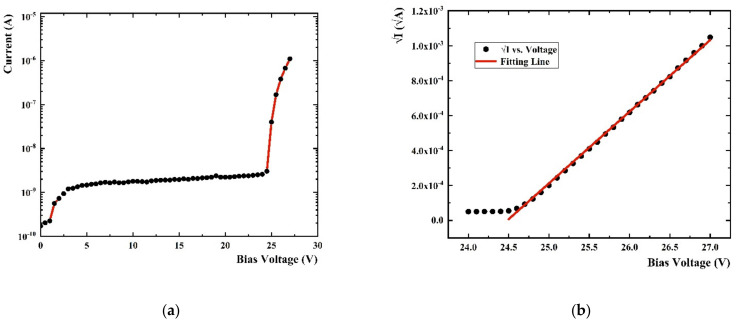
(**a**) Current vs. bias voltage plot at room temperature; (**b**) $\sqrt{I}/\sqrt{A}$ vs. bias voltage plot at room temperature.

**Figure 5:**
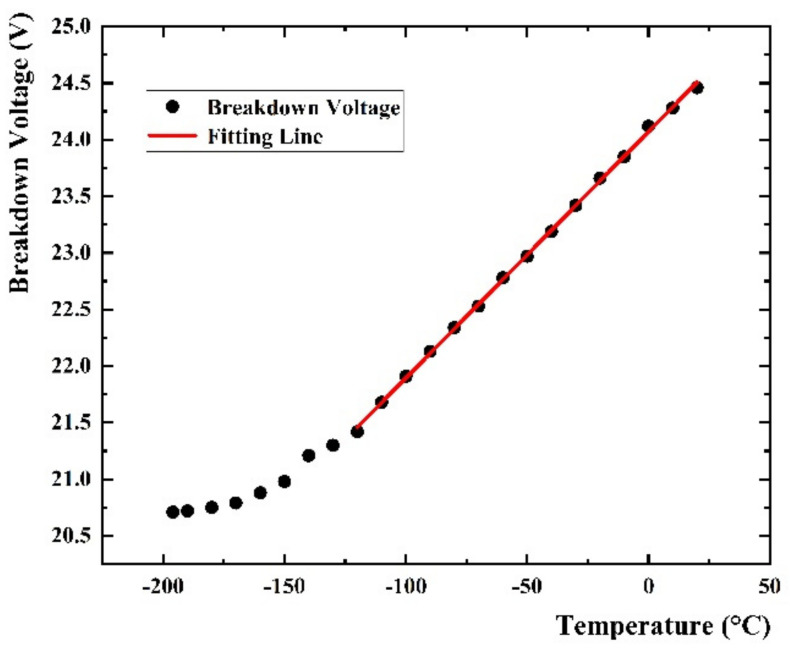
V_bd_ at different temperature, from 20 °C to −196 °C.

The breakdown voltage (V_bd_) is the bias point at which the electric field strength in the depletion region is large enough to induce a Geiger discharge. The V_bd_ point is clearly identified on a current versus voltage plot by the sudden increase in current. The I-V curve measured by the Pico-ammeter is shown in Figure 4a, where the typical curves at room temperature are shown. The Pico-ammeter shows the bias of the SiPM, and meanwhile records the currents of SiPM in the working voltage range. We set the step of working voltage change as 0.5 V, and the Pico-ammeter can display the current value varying with the voltage increasing. It can be seen that the V_bd_ of SiPM is around 25 V. Since the step size setting cannot be infinitesimally small, the accurate V_bd_ still needs to be obtained through data processing. The accurate V_bd_ is determined as the value of the voltage intercept of a straight line fit to a plot of $\sqrt{I}$ versus V [20] as shown in Figure 4b, and the fitting result at room temperature is 24.49 V which is very close to the report data (24.7 V) from ON Semiconductor’s J-Series SiPM product sheet [17]. It shows that the experimental process and data processing method are reliable and effective. The V_bd_ at different temperatures measured in this way are shown in Figure 5. We can see that the V_bd_ decreases with the decrease of temperature. This change is almost linear when the temperature is higher than −120 °C, and the change rate is approximately 0.022 V/°C. When the temperature becomes lower, the rate of V_bd_ decrease becomes slower.

The thermal vibration of the semiconductor lattice weakens with the decrease of temperature, which results in the widening of the barrier layer in the P-N junction. As such, the mean free path of the carrier movement increases and the energy obtained by the acceleration of the external electric field before colliding with the atom increases [21]. This leads to the enhancement of the chance of collision and ionization, and the probability of avalanche collision increases. Due to this condition, avalanche breakdown is more likely to occur. This is the reason that the V_bd_ becomes lower. Therefore, the V_bd_ of SiPM decreases when the environmental temperature becomes lower. This is consistent with the experimental results.

### 3.2. Dark Noise

**Figure 6:**
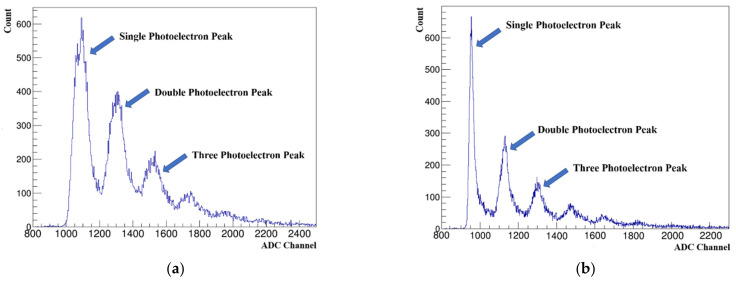
(**a**) Dark noise spectrum of SiPM at room temperature, V_over_ = 2.5 V; (**b**) dark noise spectrum of SiPM at liquid nitrogen temperature, V_over_ = 2.5 V.

SiPM has the ability to discern a single photon. Within the scope of certain intensity, the output of SiPM charge is proportional to the photon number and SiPM possess the function of the photon counter. SiPM with better performance can see clear single-photon peaks and multi-photon peaks in the spectrum of dark noise. As shown in Figure 6a, the energy spectra at room temperature and at liquid nitrogen temperature are indicated. In both cases, single-photon peaks can be seen. Since SiPM has lower dark noise at liquid nitrogen temperature, the resolution of single photon peaks is better than that at room temperature. This change shows in the peak-to-valley ratio. The resolution of single photon peak increases with increase of the peak-to-valley ratio. It can be seen from Figure 6 that the peak-to-valley ratio at room temperature is about 6 and this parameter is 12 at liquid nitrogen temperature. The single photoelectron and double photoelectron peaks can be seen clearly in the energy spectrum of SiPM. This indicates that SiPM works perfectly at liquid nitrogen temperature. 

Dark noise mainly refers to the dark current. That is, the internal current when the device works in a dark environment. DCR refers to the number of pulses output per unit of time under darkness condition. It directly influences the signal-to-noise ratio of the output signal from SiPM and heavily affects the energy resolution of the crystal scintillator detector. The dark current of SiPM is mainly composed of three parts, which are the surface leakage current, the thermal current caused by the lattice defects in the depletion zone, and the tunneling current caused by the tunnel effect. Dark counting is mainly caused by thermal effects and tunneling effects. It is unavoidable.

**Figure 7:**
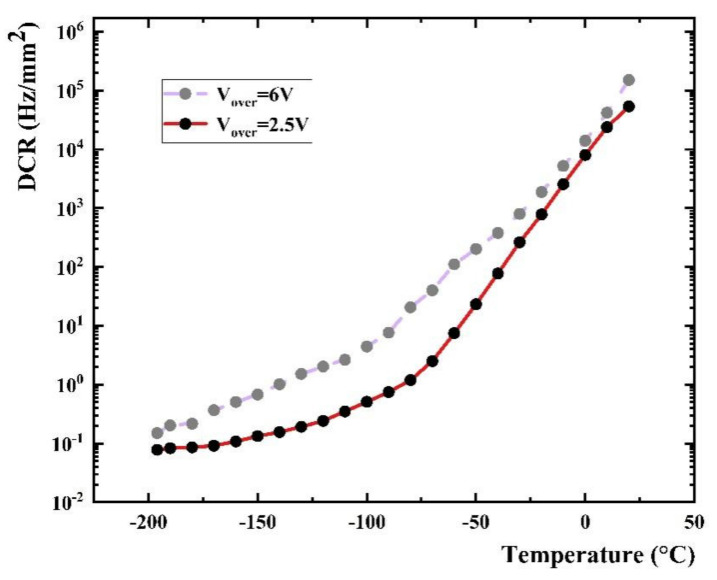
DCR vs. temperature, V_over_ = 2.5 V and V_over_ = 6 V.

Thermal current refers to the current generated by triggering an avalanche during the transition from the valence band to the conduction band of electrons. These electrons are generated by thermal excitation in the depletion zone under the action of an electric field. The calculation formula of the thermal current is as follows:

(2) $$I_{thermal-current}=AT^{2}\exp\left(-E/k_{B}T\right)$$

where *A* is a constant, *T* is the temperature, *E* is the energy range between conduction band and valence band, and *k_B_* is Boltzmann constant. It can be seen from the formula that the thermal current is related to the temperature and the current increases with the rise of temperature, and this means a higher DCR. The measured DCR versus temperature curve is shown in Figure 7. DCR dropped by six orders of magnitude from room temperature (120 kHz/mm^2^) to liquid nitrogen temperature (0.1 Hz/mm^2^). The DCR decreases rapidly with the decrease of temperature from normal temperature to −100 °C, and the decrease gradually becomes slower after −100 °C.

Tunneling current means that the electrons act in a strong electric field to free themselves from the valence band and tunnel to the conduction band, causing an avalanche. The tunneling current is mainly affected by the electric field. The dark current caused by the tunneling effect becomes greater with a higher applied voltage of SiPM, as well as DCR. As shown in Figure 7, when at the same temperature, DCR at V_over_ = 6 V is higher than it at V_over_ = 2.5 V.

**Figure 8:**
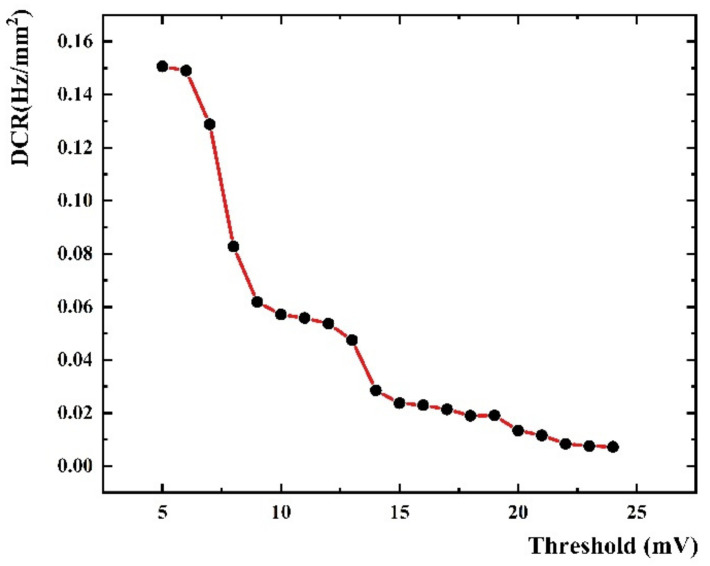
DCR vs. threshold with 6 V over voltage at liquid nitrogen temperature.

The change curve of DCR versus the threshold with 6 V over voltage at liquid nitrogen temperature is shown in Figure 8, and the obvious step structure can be seen, which corresponds to the separation structure of the dark noise spectrum. From the curves shown in Figure 8, the DCR reduces in a step-wise manner with the increase of the threshold. At the stage of low threshold, referring to the threshold of single photoelectron, DCR decreases rapidly. When the threshold range is in the two-photon to three-photon stage, DCR falls into a slower rate. The reason for this phenomenon is that, in the absence of light, most of the collected signals are single photoelectron signals. When the threshold is between single photoelectron and double photoelectrons, the single photoelectron signals get stuck, leaving only a small proportion of multi-photoelectron signals.

## 4. Conclusions

Knowing the parameters of SiPM, like the V_bd_, is a prerequisite for using SiPM at different temperatures. The V_bd_ provides a suitable operating voltage range for the use of SiPM. Dark noise will directly affect the signal-to-noise ratio, which is very important in the detection and discrimination of single-photon weak signals using SiPM.

In order to measure the performance of SiPM at liquid nitrogen temperature, we design a cooling system that can maintain the experiment environment at the setting temperature, and achieve a smooth cooling process of SiPM. The experimental results denote that the system can achieve stable control of the setting temperature ranging from the room temperature to liquid nitrogen temperature. The measured result shows that when the setting temperature is 0 °C, the temperature of the control system can fluctuate within the temperature band of −0.3 °C to 0.3 °C. Compared with the conventional temperature, we prefer the ultra-low temperature range. After fitting the experimental result, the rate of decline is less than 0.5 °C/min, which will not cause mechanical damage to the SiPM. We measure the changes of temperature near SiPM when the system temperature decreases, the experimental results show that the mass-produced J series SiPM works properly at liquid nitrogen temperature. The changes of V_bd_ and DCR with temperature are measured in detail; both of them decrease with the decrease of temperature, and the change is relatively fast at the beginning, and gradually slows down after −100 °C. DCR drops by six orders of magnitude at liquid nitrogen temperature to about 0.1 Hz/mm^2^ and this meets the requirement of a low threshold detector designing scheme. Preliminary results indicate that the J series SiPMs is one of the candidate devices for neutrino coherent detectors.

## Figures and Tables

**Table 1:** Key parameters of PMT and SiPM.

Parameter	PMT	SiPM
Working Voltage	>1000 V	30 V~80 V
Dark Count	4000~800,000	10^5^~10^6^
Photon Detection Efficiency	20%~25%	25%~70%
Gain	$10^{5}\sim 10^{6}$	$>10^{6}$

## Data Availability

Available on request.
